# Full cyclic coordinate descent: solving the protein loop closure problem in C*α *space

**DOI:** 10.1186/1471-2105-6-159

**Published:** 2005-06-28

**Authors:** Wouter Boomsma, Thomas Hamelryck

**Affiliations:** 1 Bioinformatics center, Institute of Molecular Biology and Physiology, University of Copenhagen, Universitetsparken 15, Building 10, DK-2100 Copenhagen, Denmark

## Abstract

**Background:**

Various forms of the so-called *loop closure problem *are crucial to protein structure prediction methods. Given an N- and a C-terminal end, the problem consists of finding a suitable segment of a certain length that bridges the ends seamlessly.

In homology modelling, the problem arises in predicting loop regions. In *de novo *protein structure prediction, the problem is encountered when implementing local moves for Markov Chain Monte Carlo simulations.

Most loop closure algorithms keep the bond angles fixed or semi-fixed, and only vary the dihedral angles. This is appropriate for a full-atom protein backbone, since the bond angles can be considered as fixed, while the (*φ*, *ψ*) dihedral angles are variable. However, many *de novo *structure prediction methods use protein models that only consist of C*α *atoms, or otherwise do not make use of all backbone atoms. These methods require a method that alters both bond and dihedral angles, since the pseudo bond angle between three consecutive C*α *atoms also varies considerably.

**Results:**

Here we present a method that solves the loop closure problem for C*α *only protein models. We developed a variant of Cyclic Coordinate Descent (CCD), an inverse kinematics method from the field of robotics, which was recently applied to the loop closure problem. Since the method alters both bond and dihedral angles, which is equivalent to applying a full rotation matrix, we call our method Full CCD (FCDD). FCCD replaces CCD's vector-based optimization of a rotation around an axis with a singular value decomposition-based optimization of a general rotation matrix. The method is easy to implement and numerically stable.

**Conclusion:**

We tested the method's performance on sets of random protein C*α *segments between 5 and 30 amino acids long, and a number of loops of length 4, 8 and 12. FCCD is fast, has a high success rate and readily generates conformations close to those of real loops. The presence of constraints on the angles only has a small effect on the performance. A reference implementation of FCCD in Python is available as supplementary information.

## Background

Many protein structure prediction methods require an algorithm that is capable of constructing a new conformation for a short segment of the protein, without affecting the rest of the molecule. In other words, a protein fragment needs to be generated that seamlessly closes the gap between two given, fixed end points. This problem is generally called the *loop closure problem*, and was introduced in a classic paper by Go and Scheraga more than 30 years ago [1]. It has been the continued subject of intensive research over many years due to its high practical importance in structure prediction.

The loop closure problem arises in at least two different structure prediction contexts. In homology modelling, it is often necessary to rebuild certain loops that differ between the protein being modelled and the template protein [2]. The modelled loop needs to bridge the gap between the end points of the template's loop.

In *de novo *prediction, *local resampling *or *local moves *can be considered as a variant of the loop closure problem. Typically, the conformation of a protein segment needs to be changed without affecting the rest of the protein as a sampling step in a Markov Chain Monte Carlo (MCMC) procedure [3]. In both homology and *de novo *structure prediction, the problem is however essentially the same.

The classic article by Go and Scheraga [1] describes an analytical solution to finding all possible solutions for a protein backbone of three residues. In this case, the degrees of freedom (DOF) comprise six dihedral angles, ie. the backbone's (*φ*, *ψ*) angles. Another approach is to use a fragment library derived from the set of solved protein structures, and look for fragments or combinations of fragments that bridge the given fixed ends [4-6]. More recently, the loop closure problem has been tackled using algorithms borrowed from the field of robotics, in particular inverse kinematics methods [7-9]. Still other methods use various Monte Carlo chain perturbation approaches, often combined with analytical methods [10,3,12]. A good overview of loop closure methods and references can be found in Kolodny *et al. *(2005) [6].

Most methods assume that one is working with a full-atom protein backbone with fixed bond angles and bond lengths, so the DOF consist solely of the backbone's (*φ*, *ψ*) angles. However, in many cases not all the atoms of the protein backbone are present in the model. In particular, a large class of structure prediction, design and *in silico *folding methods makes use of drastically simplified models of protein structure [13,14].

A protein structure might for example be represented by a chain of C*α *atoms or a chain of virtual atoms at the centers of mass of the side chain atoms [15]. In these models, there is obviously no full-atom model of the protein's backbone available.

**Figure 1:**
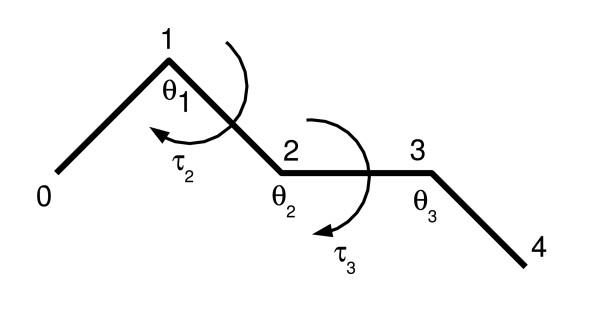
A protein segment's C*α *trace. The C*α *positions are numbered, and the pseudo bond angles *θ *and pseudo dihedrals *τ *are indicated. The segment has length 5, and is thus fully described by two pseudo dihedral and three pseudo bond angles.

In the case of C*α*-only models, the structure can be described as a sequence of pseudo bonds, pseudo angles *θ *and pseudo dihedral angles *τ *[16]. Here, the term 'pseudo' indicates that the consecutive C*α*'s are not actually connected by chemical bonds. As in the case of the protein's backbone, the pseudo bond lengths can be considered fixed (typically 3.8 Å). In contrast, the pseudo bond angles between three consecutive C*α *atoms are most definitely not fixed, but vary between 1.4 and 2.7 radians. Hence, a C*α*-only model of *N *residues can be represented by a sequence of *N *- 2 pseudo bond angles *θ *and *N *- 3 pseudo dihedral angles *τ *(Figure 1).

Most inverse kinematics approaches assume that the DOF consist only of dihedral angles, and keep the bond angles fixed or semi-fixed. Hence, they cannot be readily applied to the C*α*-only case without restricting the search space unnecessarily. In principle, fragment library based methods would apply, but here the problem of data sparsity arises [17,18]. Often, no suitable fragments can be found if the number of residues between the fixed ends becomes too high.

In order to solve the loop closure problem in C*α *space, we extend a particularly attractive approach that was recently introduced by Canutescu & Dunbrack [8]. The algorithm is called Cyclic Coordinate Descent (CCD), and like many other loop closure algorithms it derives from the field of robotics [19]. As pointed out by Canutescu & Dunbrack, the CCD algorithm is meant as a black box method that generates plausible protein segments that bridge two given, fixed endpoints. The final choice is typically made based upon the occurrence of steric clashes, applicable constraints (for example side chain conformations) and evaluation of the energy.

The CCD algorithm does not directly generate conformations that bridge a given gap, but alters the dihedral angles of a given starting segment that already overlaps at the N-terminus such that it also closes at the C-terminus. The starting segment can be generated in many ways, for example by using a fragment library derived from real structures or by constructing random artificial fragments with reasonable conformations. Surprisingly, most protein loops can be closed efficiently by CCD starting from artificial loops constructed with random (*φ*, *ψ*) dihedral angles [8].

The CCD algorithm alters the (*φ*, *ψ*) dihedral angles for every residue in the segment in an iterative way. In each step, the RMSD between the chain end and the overlap is minimized by optimizing one dihedral angle. Because only one dihedral angle is optimized at a time, the optimal rotation can be calculated efficiently using simple vector arithmetic.

The list of advantages of CCD is impressive: it is conceptually simple and easy to implement, computationally fast, very flexible (ie. capable of incorporating various restraints and/or constraints) and numerically stable. Therefore, we decided to adopt the CCD algorithm for use with C*α*-only models. Here, we describe a new version of CCD that optimizes both dihedral angles and bond angles, while maintaining all the advantages of the CCD method. We call our method Full Cyclic Coordinate Descent (FCCD), where "Full" indicates that both dihedral angles and bond angles are optimized, while only the bond lengths remain fixed. At the heart of the FCCD method lies a procedure to superimpose point sets with minimal Root Mean Square Deviation (RMSD), based on singular value decomposition. As is the case for the CCD algorithm, FCCD is not a modelling method in itself. Rather, it can be used as a method to generate possible conformations that can be evaluted using some kind of energy function.

To test the algorithm, we selected random segments from a protein structure database, and evaluated the efficiency of closing the corresponding gaps starting from artificial segments with protein-like (*θ*, *τ*) angles. We show that FCCD is both fast and successful in solving the loop closure problem, even in the presence of angle constraints. Conformations close to those of real protein loops are readily generated. Finally, we discuss possible applications of the FCCD algorithm, and mention some possible disadvantages.

## Results and discussion

### Overview of the FCCD algorithm

**Figure 2:**
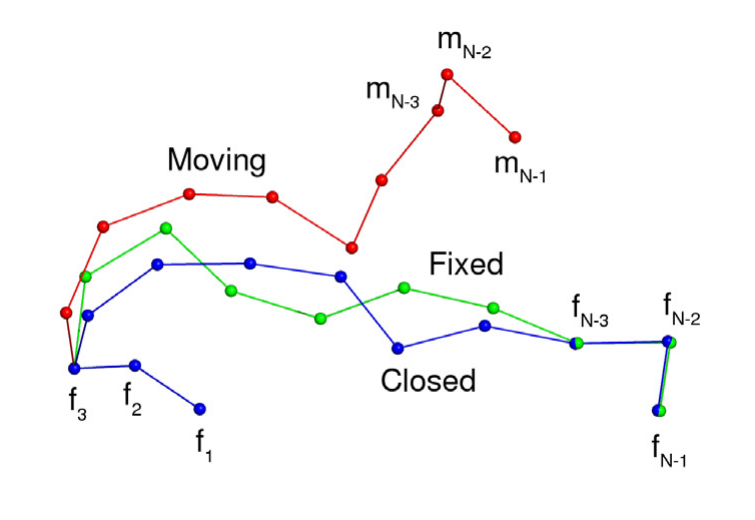
The action of the FCCD algorithm in C*α *space. The C*α *traces of the moving, fixed and closed segments are shown in red, green and blue, respectively. The C*α *atoms are represented as spheres. The labels *f*_0_, *f*_1 _and *f*_2 _indicate the three fixed vectors at the N-terminus that are initially common between the fixed and moving segments. The loop is closed when the three C-terminal vectors of the moving segment (labelled *m*_*N*-3_, *m*_*N*-2_, *m*_*N*-1_) superimpose with an RMSD below the given threshold on the three C-terminal vectors of the fixed segment (labelled (*f*_*N*-3_, *f*_*N*-2_, *f*_*N*-1_). This figure and Figure 3 were made with PyMol .

Figure 2 illustrates the essence of the FCCD algorithm, and Table 3 provides detailed pseudo code. Here we define some of the terms that will be used throughout the article, and provide a high level overview of the FCCD algorithm.

The *fixed segment *is a list of C*α *vector positions that specifies the gap that needs to be bridged. Only the first and last three C*α *positions, with corresponding vectors (*f*_0_, *f*_1_, *f*_2_) and (*f*_*N*-3_, *f*_*N*-2_, *f*_*N*-1_) are relevant. We will call these two sets of vectors the *N- and C-terminal overlaps*, respectively. The *moving segment *is a list of C*α *position vectors that will be manipulated by the FCCD algorithm to bridge the gap. The *closed segment *is the moving segment after its pseudo bond angles and pseudo dihedral angles were adjusted to bridge the N- and C-terminal overlaps of the fixed segment. The vectors describing the positions of the C*α *atoms in a segment of *N *residues are labelled from 0 to *N *- 1.

Initially, the first three vectors of the moving loop coincide with the first three vectors of the fixed segment, while the last three vectors are conceivably reasonably close to the last three vectors of the fixed loop. This last condition is however not very critical. The moving segment can be obtained using any algorithm that generates plausible C*α *fragments, including deriving them from real protein structures. The fixed segment is typically derived from a real protein of interest, or a model in an MCMC simulation.

The FCCD algorithm changes the pseudo bond angles and pseudo dihedral angles of the moving loop in such a way that the RMSD between the last three vectors of the moving loop (*m*_*N*-3_, *m*_*N*-2_, *m*_*N*-1_) and the last three vectors of the fixed loop (*f*_*N*-3_, *f*_*N*-2_, *f*_*N*-1_) is minimized, thereby seamlessly closing the gap.

Note that we assume that the last three vectors of the moving and fixed segments can be superimposed with an RMSD of 0.0 Å (see Figure 2). In other words, the first and last pseudo bond angles in both segments are equal. It is however perfectly possible to use segments with different pseudo bond angles at these positions. Since the final possible minimum RMSD will be obviously greater than 0 in this case, the RMSD threshold needs to be adjusted accordingly.

The algorithm proceeds in an iterative way. In each iteration, a vector *m*_*i *_in the moving segment is chosen that will serve as a center of rotation. This chosen center of rotation will be called the pivot throughout this article. Then, the rotation matrix that rotates (*m*_*N*-3_, *m*_*N*-2_, *m*_*N*-1_) on (*f*_*N*-3_, *f*_*N*-2_, *f*_*N*-1_) around the pivot and resulting in minimum RMSD is determined, and applied to all the vectors *m*_*j *_downstream *i *(with *i *<*j *<*N*). In the next iteration, a new pivot is chosen, and the procedure is repeated. The vectors in the chain can be traversed linearly, or they can be chosen at random in each iteration. The difference between FCCD and CCD is that the latter applies a general rotation to the chain using an atom in the chain as a pivot, while the former only applies a rotation around a single axis. The process is stopped when the RMSD falls below a given threshold.

Finding the optimal (with respect to the RMSD) rotation matrix corresponds to finding one optimal pseudo bond angle and pseudo dihedral angle pair. We define *θ*_*i *_as the bond angle of the vectors *m*_*i*-1_, *m*_*i*_, *m*_*i*+1 _and *τ*_*i *_as the dihedral angle of the vectors *m*_*i*-2_, *m*_*i*-1_, *m*_*i*_, *m*_*i*+1 _(see Figure 1 and [16]). These definitions have the intuitive interpretation that altering (*θ*_*i*_, *τ*_*i*_) changes the positions of all C*α*'s downstream from position *i*. Conversely, using pivot *m*_*i *_and applying a rotation matrix to all the positions downstream from position *i *corresponds to changing pseudo bond angle *θ*_*i*_ and pseudo dihedral angle *τ*_*i*_.

For a segment of *N *C*α*'s (with *N *> 3), the pseudo angles range from *θ*_1 _to *θ*_*N*-2 _and the pseudo dihedrals range from *τ*_2 _to *τ*_*N*-2_. Since the first and last bond angles of the moving segment are fixed, the pivot points range from position 2 to position *N *- 3 (with *N *> 4). The pseudo bond angle and pseudo dihedral angle pairs thus range from (*θ*_2_, *τ*_2_) to (*θ*_*N*-3_, *τ*_*N*-3_).

Finding the optimal rotation matrix with respect to the RMSD of the C-terminal overlaps can be efficiently solved using singular value decomposition, as described in detail in the following section.

### Finding the optimal rotation

In this section we discuss solving the following subproblem arising in the FCCD algorithm: given a chosen pivot point *i *in the moving segment, find the optimal (*θ*_*i*_, *τ*_*i*_) pair that minimizes the RMSD between the last three C*α *vectors in the moving segment and the last three C*α *vectors in the fixed segment. Recall that the (*θ*_*i*_, *τ*_*i*_) pair at position *i *corresponds to the pseudo bond angles and pseudo dihedral angles defined by vectors *m*_*i*-1_, *m*_*i*_, *m*_*i*+1 _and *m*_*i*-2_, *m*_*i*-1_, *m*_*i*_, *m*_*i*+1 _respectively.

Finding the optimal (*θ*_*i*_, *τ*_*i*_) pair simply corresponds to finding the optimal rotation matrix using C*α *position *i *as the center of rotation (see Figure 2). This reformulated problem can be solved by a variant of a well known algorithm to superimpose two point sets with minimum RMSD which makes use of singular value decomposition [20,21]. Below, we describe this adapted version of the algorithm.

First, the C-terminal overlaps of the moving and the fixed segment need to be translated to the new origin that will be used as pivot for the optimal rotation. This new origin is the pivot vector *m*_*i*_ at C*α *position *i *in the moving segment. The new vector coordinates of the moving and the fixed segments are put in two matrices (respectively *M *and *F*), with the coordinates of the vectors positioned column wise:

*M *= [*m*_*N*-3 _- *m*_*i *_| *m*_*N*-2 _- *m*_*i *_| *m*_*N*-1 _- *m*_*i*_]

*F *= [*f*_*N*-3 _- *m*_*i *_| *f*_*N*-2 _- *m*_*i *_| *f*_*N*-1 _- *m*_*i*_]

Then, the correlation matrix Σ is calculated using *M *and *F *:

Σ = *FM*^*T*^

Any real *n *× *m *matrix *A *can be written as the product of an orthogonal *n *× *n *matrix *U*, a diagonal *n *× *m *matrix *D *and an orthogonal *m *× *m *matrix *V*^*T *^[22]. Such a factorization is called a s*ingular value decomposition *of *A*. The positive diagonal elements of *D *are called the *singular values*. Hence, Σ can be written as:

Σ = *UDV*^*T*^

The optimal rotation Γ is then calculated as follows:

Γ = *USV*^*T*^

The value of the diagonal 3 × 3 matrix *S *is determined by the product det(*U*)det(*V*^*T*^), which is either 1 or -1. If this product is -1 then *S *= diag(1, 1, -1), else *S *is the 3 × 3 unit matrix. The matrix *S *ensures that Γ is always a pure rotation, and not a rotation-inversion [21].

In order to apply to all the vectors that are downstream from the pivot point i, these vectors are first translated to the origin of the rotation (ie. pivot point *m*_*i*_), left multiplied by Γ and finally translated back to the original origin:



where *i *<*j *<*N*.

### Adding angle constraints to FCCD

It is straightforward to constrain the (*θ*, *τ*) angles to a given probability distribution. For each rotation matrix Γ, the resulting new pseudo bond angles and dihedral angles can easily be calculated. The new angles can for example be accepted or rejected using a simple rejection sampling Monte Carlo scheme, comparing the probabilities of the previous pair (*θ*^*prev*^, *τ*^*prev*^) with that of the next pair (*θ*^*next*^, *τ*^*next*^). If *P *(*θ*^*next*^, *τ*^*next*^) > *P *(*θ*^*prev*^, *τ*^*prev*^) the change is accepted, otherwise it is accepted with a chance proportional to *P *(*θ*^*next*^, *τ*^*next*^) / *P *(*θ*^*prev*^, *τ*^*prev*^). A similar approach was used by Canutescu & Dunbrack [8], and we describe its performance in combination with FCCD in the following section.

More advanced methods could take the probability of the sequence of angles into account as well, for example using a Hidden Markov Model of the backbone [23]. The pseudo code in Table 3 illustrates accepting/rejecting rotations using an unspecified 'accept' function, whose details will depend on the application.

### FCCD's performance

In order to evaluate the general efficiency of the method, we selected random fragments of various sizes from a representative database of protein structures, and used these fragments as fixed segments. Hence, the evaluation described below is not limited to loops, but extends to random protein segments. This is a relevant test, since local moves in a typical MCMC simulation are indeed performed on random segments.

The fixed segments were sampled from a dataset of fold representatives (see Methods). First we selected a random fold representative, and subsequently extracted a random continuous fragment of suitable length. The lengths varied from 10 to 30 with a step size of 5. It should be noted that the length of the segment here refers to the number of C*α *atoms between the ends that need to be bridged.

The moving segments were generated using random dihedral and bond angles in regions accessible to proteins (see previous section). This was done by sampling the (*θ*_*i*_, *τ*_*i*_) pairs according to a probability distribution derived from a set of representative protein structures (see Methods). The bond length was fixed at 3.8 Å, in tune with the consensus C*α*-C*α *distance in protein structures. The last bond angle in the moving segment was chosen equal to the last bond angle in the fixed loop to make a final RMSD of 0.0 Å possible. The RMSD threshold was 0.1 Å. The maximum number of iterations was set to 1000, where one iteration is a sweep over all positions. We ran the FCCD program on 1000 different fixed segments. Table 1 summarizes the results.

**Table 1:** Performance of the FCCD algorithm for various segment lengths. The first and second number in columns 2–4 refer to unconstrained and constrained FCCD, respectively. Columns 2 and 3 respectively show the average time and number of iterations needed for closing a single segment successfully. The percentage of loops successfully closed in under 1000 iterations is shown in the last column.

Segment length	Average time (ms)	Average iterations	% Closed
5	4.5/51.7	14.0/27.0	99.90/86.50
10	5.2/28.3	10.5/16.8	99.40/98.20
15	5.6/28.6	7.8/12.1	99.60/99.40
20	6.2/27.1	6.3/9.0	99.80/99.40
25	7.6/31.7	5.5/7.6	99.00/99.90
30	7.1/31.0	4.4/6.3	99.70/99.40

A first observation is the effect of the angle constraints. These slow down FCCD with a factor of 10 for small segments (5 residues) and roughly a factor of 5 for larger segments (10 residues or more). Nonetheless FCCD including constraints remains quite speed efficient: small five residue segments are on average closed in about 50 ms, while larger segments (from 10 to 30 residues) are closed considerably faster (on average in about 30 ms). The explanation for this is of course that it is easier to close large segments because they have more DOF. Hence, FCCD, like CCD, is fast and easily handles large segments efficiently.

Overall, the success rate of FCCD is excellent, and very little affected by constraints. For 5 residue segments, adding constraints diminishes the number of successfully closed segments from 99.9% to 86.5%. This effect is however much less pronounced for larger segments: more than 98% percent of the moving/fixed segment pairs can be successfully closed. In short, FCCD is both speed efficient and has a high success rate, even in the presence of constraints.

### Evaluation of FCCD's sampling space

Does FCCD potentially generate realistic protein conformations? FCCD could be used to propose possible conformations that are subsequently evaluated by an energy function. In this context, it is of course imperative to generate realistic conformations. To answer this question, we evaluate FCCD's ability to generate closed segments that are close to real protein loops. We used 30 real loops with lengths of 4, 8 and 12 residues as fixed segments. The loop length refers to the number of residues between the N- and C-terminal overlaps.

**Figure 3:**
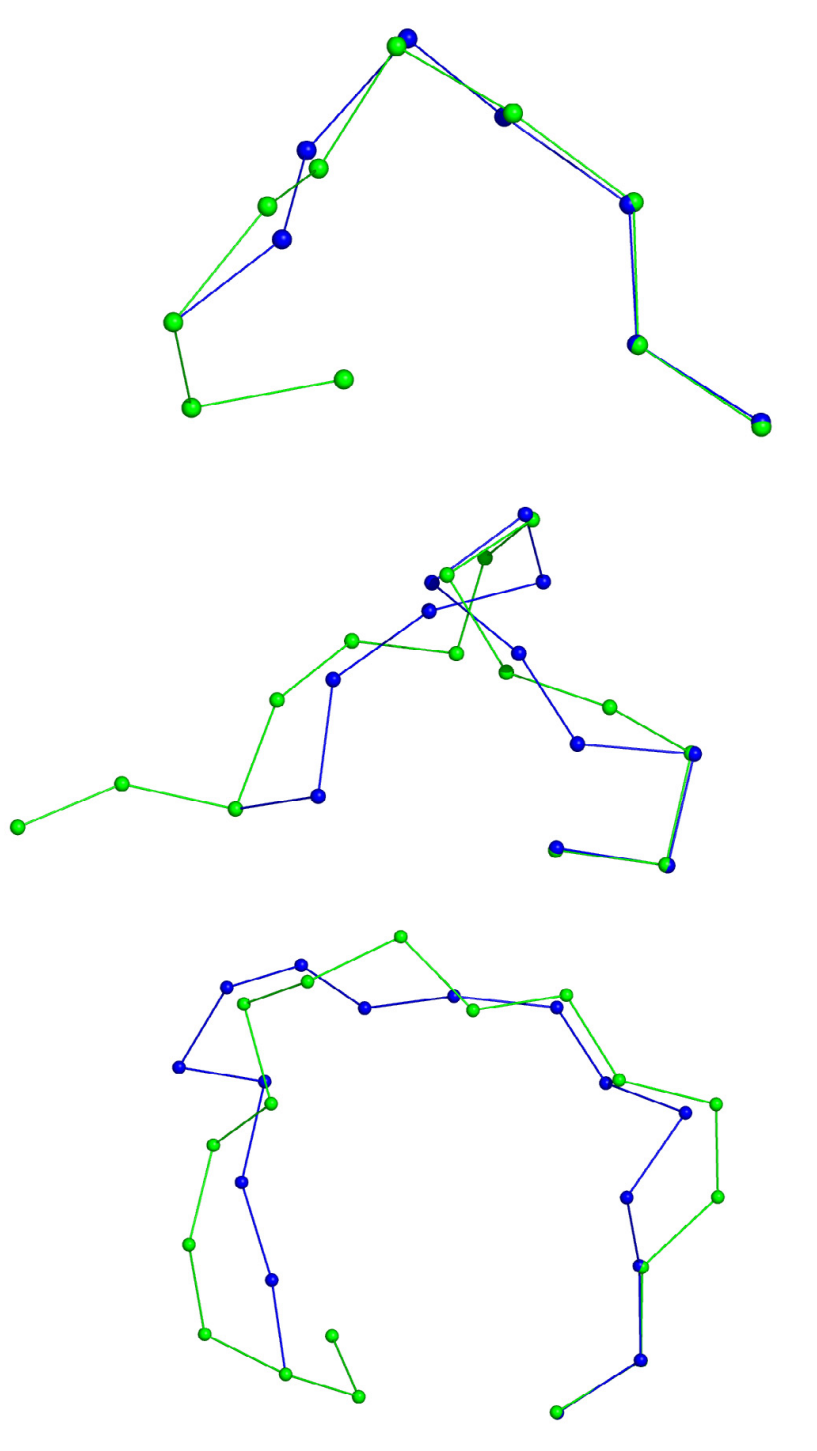
Loops generated by FCCD (blue) that are close to real protein loops (green). The loops with lowest RMSD to a given loop of length 4 (top), 8 and 12 (bottom) are shown (loops 1qnr, A, 195–198, 3chb, D, 51–58 and 1ctq, A, 26–37). The N- terminus is at the left hand side.

FCCD was applied using (*θ*, *τ*) constraints and an RMSD threshold of 0.1 Å. The maximum number of iterations was set to 1000. For each loop, we attempted to generate closed segments from 1000 random moving segments within the allowed number of iterations. The moving segments were generated as described in the previous section. For all 30 loop cases, we then identified the closed segment that resembled the input loop best as judged by the RMSD. For the calculation of the RMSD, we included the N-and C-terminal overlaps. The results are shown in Table 2, and the best fitting loops for each loop size are shown in Figure 3.

**Table 2:** Minimum RMSD (out of 1000 tries) between a fixed segment derived from a protein structure and a closed segment generated by FCCD. The length of the loops is shown between parentheses in the upper row.

Loop (4)	RMSD	Loop (8)	RMSD	Loop (12)	RMSD
1dvj, A, 20–23	0.59	1cru, A, 85–92	2.31	1cru, A, 358–369	3.37
1dys, A, 47–50	0.67	1ctq, A, 144–151	2.22	1ctq, A, 26–37	2.40
1egu, A, 404–407	0.61	1d8w, A, 334–341	2.04	1d4o, A, 88–99	3.20
1ej0, A, 74–77	0.61	1ds1, A, 20–27	2.20	1d8w, A, 43–54	2.74
1i0h, A, 123–126	0.73	1gk8, A, 122–129	2.20	1ds1, A, 282–293	3.16
1id0, A, 405–408	0.66	1i0h, A, 145–152	2.42	1dys, A, 291–302	2.90
1qnr, A, 195–198	0.54	1ixh, 106–113	1.98	1egu, A, 508–519	3.06
1qop, A, 44–47	0.58	1lam, 420–427	2.16	1f74, A, 11–22	3.12
1tca, 95–98	0.76	1qop, B, 14–21	2.17	1q1w, A, 31–42	3.04
1thf, D, 121–124	0.56	3chb, D, 51–58	1.97	1qop, A, 175–186	2.97

Average RMSD	0.63	Average RMSD	2.17	Average RMSD	3.00

**Table 3:** 

*maxit *= maximum number of iterations
*moving *= *N *× 3 matrix of C*α *positions in moving segment
*fixed *= *N *× 3 matrix of C*α *positions in fixed segment
*threshold *= desired minimum RMSD
*N *= length of the segments
*M *= 3 × 3 matrix (centered coordinates along columns)
*F *= 3 × 3 matrix (centered coordinates along columns)
*S *= diag(1, 1, -1)
**repeat ***maxit*:
# Start iteration over pivots
**for ***i ***from **2 **to ***N*-3:
*pivot *= *moving*[*i*,:]
**# **Make pivot point origin
**for ***j ***from **0 **to **2:
*M *[:,*j*] = *moving *[*N*-3+*j*,:]-*pivot*
*F *[:,*j*] = *fixed *[*N*-3+*j*,:]-*pivot*
**# **Find the rotation Γ that minimizes RMSD
Σ = *FM*^*T*^
*U*, *D*, *V*^*T *^= **svd**(Σ)
**# **Check for reflection
**if **det(*U*)det(*V*^*T*^)<0:
*U *= *US*
Γ = *UV*^*T*^
**# **Evaluate and apply rotation
**if **accept(Γ):
**# **Apply the rotation to the moving segment
**for ***j ***from ***i*+1 to *N*-1:
* moving *[*j*,:] = Γ (*moving *[*j*,:]-*pivot*)+*pivot*
*rmsd *= calc_rmsd(*moving *[*N*-3,:], *fixed *[*N*-3,:])
**# **Stop if RMSD below threshold
**if ***rmsd*<*threshold*:
** return ***moving*, *rmsd*
# Failed: RMSD threshold not reached before maxit
**return **0

The accept function rejects or accepts the proposed rotation, based on the resulting (*θ*, *τ*) pair. The svd function performs singular value decomposition, and calc_rmsd calculates the RMSD between two lists of vectors.

**Table 4:** SABMark identifiers of the 236 structures used as fold representatives

1ew6a_	1ail__	1l1la_	1kid__	1n8yc1	1gzhb1	1e5da1	1ep3b2	1ihoa_	1m0wa1
1dhs__	1gpua2	2lefa_	1nsta_	1eaf__	1iiba_	1d5ra2	1foha3	1gpua3	1crza2
3pvia_	1i6pa_	1e4ft1	1kx5d_	2pth__	1lu9a2	1dkla_	1fsga_	1m2oa3	2dpma_
1ajsa_	1fxoa_	3tgl__	1bx4a_	1mtyg_	1duvg2	1qopb_	1iata_	1k2yx2	1f0ka_
1ayl_1	1toaa_	8abp__	1nh8a1	1bi5a2	2mhr__	1a2pa_	3lzt__	1dkia_	1e7la2
1bf4a_	1bb8__	1kpf__	1mu5a2	1lfda_	1gpea2	1jqca_	1a2va2	1jfma_	1ll7a2
1cjxa1	1lo7a_	1fm0e_	1fs1b2	1o0wa2	1dtja_	1k0ra3	1evsa_	1jpdx2	1qd1a1
1d5ya3	1h3fa2	1iq0a3	1tig__	1xxaa_	1ck9a_	1gyxa_	1e5qa2	1ivsa2	1qbea_
3grs_3	1f08a_	1c7ka_	1lkka_	1dq3a3	1uox_1	12asa_	1bob__	1m4ja_	1dv5a_
1f5ma_	1k2ea_	1ei1a2	1jdw__	1ln1a_	2pola2	1f0ia1	1rl6a1	1fvia2	1j7la_
1is2a1	1e8ga2	1qr0a1	2dnja_	1kuua_	1qh5a_	1ii7a_	1b8pa2	1j7na3	1chua3
1f00i3	1grj_1	1nkd__	1mwxa3	1jp4a_	1ih7a2	1eula2	1gnla_	1maz__	2por__
4htci_	1es7b_	1tocr1	1d1la_	1fd3a_	1i8na_	1h8pa1	4sgbi_	1fltv_	1quba1
1d4va3	1tpg_2	1iuaa_	1fv5a_	1mdya_	1zmec1	1fjgn_	1eska_	1i50i2	1fbva4
1dmc__	1e53a_	1ezvb1	1jeqa1	1k3ea_	1rec__	1lm5a_	1k82a1	1jaja_	1m0ka_
1c0va_	1kqfc_	1ocrk_	1h67a_	2cpga_	1ljra1	1brwa1	1hs7a_	2cbla2	1jmxa2
1hyp__	1cuk_2	1ecwa_	1l9la_	1g7da_	1jkw_1	1dgna_	1iqpa1	1pa2a_	1ko9a1
1f1za1	1ks9a1	2sqca2	1d2ta_	1h3la_	1wer__	1b3ua_	1n1ba2	1poc__	1e79i_
1m1qa_	1enwa_	1g4ma1	1e5ba_	1qhoa2	1kv7a2	1l4ia2	1c8da_	1amm_1	1ca1_2
1phm_2	1d7pm_	1jjcb2	1flca1	1gr3a_	1mjsa_	1a8d_1	1lf6a2	1fqta_	1jb0e_
1jh2a_	1lcya1	1mgqa_	1hcia1	1b3qa2	1jlxa1	1dar_1	1exma2	1ejea_	1agja_
1e79d2	2rspa_	1h0ha1	1gtra1	2erl__	1btn__	1lf7a_	1jmxa5	1crua_	1m1xa4
1hx0a1	1goia1	1ciy_2	1daba_	3tdt__	1gg3a1	1pmi__	1bdo__	1h3ia2	1gppa_
1f39a_	1k6wa1	1jqna_	1lu9a1	1m6ia1	1o94a3				

It is clear that FCCD readily generates closed segments that are reasonably close to the real loops, with an average RMSD of about 0.6, 2.2 and 3.0 Å for loops of 4, 8 and 12 residues, respectively. The highest minimum RMSD values for these loop lengths are 0.76, 2.42 and 3.37 Å, respectively, indicating that FCCD in general can come up with a reasonably close conformation. Using more initial moving segments will obviously increase the chance of encountering a close conformation. Additionally, one can also expect an even better performance with a more refined way to constrain the (*θ*, *τ*) angles.

## Conclusion

In this article, we introduce an algorithm that solves the loop closure problem for C*α *only protein models. The method is conceptually similar to the CCD loop closure method introduced by Canutescu and Dunbrack [8], but optimizes dihedral and bond angles simultaneously, while the former method only optimizes one angle at a time. At the heart of the method lies a modified algorithm to superimpose point sets with minimum RMSD, based on singular value decomposition [20,21].

The algorithm is fast, numerically stable and leads to a solution for the great majority of loop closure problems studied here. Importantly, the method remains efficient even in the presence of constraints on the dihedral and bond angles. FCCD readily handles large gaps, and potentially generates realistic conformations. Compared to other loop closure methods, FCCD is surprisingly easy to implement provided a function is available to calculate the singular value decomposition of a matrix.

A possible disadvantage is that FCCD has a tendency to induce large changes to the pseudo angles at the start of the moving segment while angles near the end are less affected, which is also the case for CCD [8]. This can for example be avoided by selecting the pivot points in a random fashion, or by limiting the allowed change in the angles per iteration. Occasionally the method gets stuck, which can be avoided by incorporating stochastic changes away from the encountered local minimum. One can also simply try again with a new random moving segment. We believe that CCD and FCCD despite these disadvantages are among the most efficient loop closure algorithms currently available.

The FCCD algorithm proposed here has great potential for use in structure prediction methods that only make use of C*α *atoms, or that otherwise do not include all backbone atoms [15,13,14]. FCCD could be used for example to implement local moves in a MCMC procedure. The moving segments could be derived from a fragment database or generated from a probabilistic model of the protein backbone. The latter model could range from a primitive probability distribution over allowed (*θ*, *τ*) angle pairs like we used here to a Hidden Markov Model that also models the sequence of (*θ*, *τ*) angle pairs.

We are planning to use the FCCD algorithm in combination with a sophisticated probabilistic model of the protein's backbone, which will steer both the generation of the initial moving loop and the acceptance/rejection of the angles. The performance of FCCD in this context will be the subject of a future publication.

## Methods

### Implementation

The FCCD algorithm was implemented in C, using the LAPACK [24] function dgesvd for the calculation of the singular value decomposition. Handling PDB files and calculating the (*θ*, *τ*) angles [16] was done using Biopython's Bio.PDB module [25]. We used a 2.5 GHz Pentium processor to calculate the benchmarks. A reference implementation of FCCD in Python is available as supplementary information.

### Structure databases

For the calculation of the (*θ*, *τ*) probability distribution and the generation of random protein fragments, we used the SABMark 1.63 Twilight Zone database [26]. SABMark Twilight Zone contains 2230 high quality protein structures, divided over 236 different folds. All protein pairs have a BLAST E-value below 1, and thus presumably belong to different superfamilies. A dataset of fold representatives was generated by selecting a single structure at random for each fold (see Table 4).

The loops used to evaluate FCCD's sampling space were derived from Canutescu & Dunbrack [8]. We shifted two loops (1d8w, A, 46–57 and 1qop, A, 178–189) by three residues to ensure that all loops had three flanking residues on each side.

### Calculation of the (*θ*, *τ*) probability distribution

The bond angle *θ *was subdivided in 18 bins and the dihedral angle *τ *in 36 bins, in both cases starting at 0 degrees and with a bin width of 10 degrees. All (*θ*, *τ*) angles were extracted from all structures in the SABMark Twilight Zone database that consisted of a polypeptide chain without breaks. In total, 257534 angle pairs were extracted. Each such (*θ*, *τ*) angle pair was assigned to a bin pair, and the number of angle pairs assigned to each bin pair was stored in a 18 × 36 count matrix. Finally, the normalized count matrix was used to assign a probability to any given (*θ*, *τ*) angle pair.

## List of abbreviations

• CCD: Cyclic Coordinate Descent

• DOF: Degrees Of Freedom

• FCCD: Full Cyclic Coordinate Descent

• MCMC: Markov Chain Monte Carlo

• RMSD: Root Mean Square Deviation

## Authors' contributions

TH conceived the FCCD algorithm. WB implemented FCCD in the C language, and introduced various refinements and optimizations. Both authors read and approved the article.

## Supplementary Material

Additional File 1 The file FCCD.py contains an implementation of the FCCD algorithm. The program was implemented in the interpreted, object oriented language Python . The Numeric Python package , a Python module that implements many advanced mathematical operations efficiently in C and FORTRAN, provided implementations of singular value decomposition and various matrix operations. In addition, the Biopython toolkit, a set of Bioinformatics modules implemented in Python, was used to represent atomic coordinates as vector objects [25]. The core of the FCCD implementation comprises only 50 lines of Python code. Numeric Python and Biopython (version 1.4b) are needed to execute the sample code. Click here for file
